# Air volume flow rate optimization of the guide vanes in an axial flow fan based on DOE and CFD

**DOI:** 10.1038/s41598-023-31666-w

**Published:** 2023-03-17

**Authors:** Fanbao Chen, Guanzhang Zhu, Danyang Xi, Bin Miao

**Affiliations:** 1 School of Safety Engineering, China University of Mining and Technology, Xuzhou, 221116 China; 2 Beijing Tianma Intelligent Control Technology Co., Ltd., Beijing, 101320 China; 3 School of Resources and Geosciences, China University of Mining and Technology, Xuzhou, 221116 China; 4 College of Resources, Shandong University of Science and Technology, Tai’an, 271019 China; 5 National Engineering Laboratory for Coalmine Backfilling Mining, Shandong University of Science and Technology, Tai’an, 271019 China

**Keywords:** Aerospace engineering, Mechanical engineering

## Abstract

The unreasonable design of guide vanes in the axial fan could have negative effects. In order to enhance the performance, the relationship between the air volume flow rate of the selected axial fan and geometric parameters of guide vanes is firstly analysed by DOE and CFD, and optimal parameters are found by the Gaussian Process method. Results show that the number and total chord of guide vanes have a nonlinear effect on the air volume flow, and the total chord of vanes is the main factor in affecting calculation results. For the particular configuration studied here, the optimal design of guide vanes shows that lessening the chord of vanes by 38 mm and increasing the number of the vanes to 18 could produce more airflow under the same rotation speed.

## Introduction

The axial flow fan, an important mechanical device in production and life, is widely used in daily life and industrial production. In China, the power consumption of pumps and fan equipment accounts for more than half of the country's power generation, and the operating efficiency of fan equipment in actual production and life is about 40–60%, far lower than the regulations. Effective improvement of fan efficiency can reduce electricity consumption, which has great significance for energy conservation and emission reduction and environmental protection^1^.

The aerodynamic characteristics of axial flow fans are complicated, and the main influencing factors are as follows: blade number, shape, blade installation angle, blade tip clearance size, hub tip ratio, collector, diffuser, etc. Many scholars have carried out simulation analysis on the airflow inside the axial flow fan through the CFD (Computational Fluid Dynamics) method and obtained a lot of results. For example, Vad^2^ found that the vaneless compression rotors and the performance of axial fans could be effectively improved by blade forward tilt and blade forward sweep. Hurault et al.^3^ studied the effects of the axial flow fan sweep on the airflow by CFD and experiments and found that the turbulent kinetic energy downstream of the fan is highly affected by the sweep. Aykut and Ünverdi^4^ carried out a CFD simulation of a six-blade axial fan and compared the results of the simulation with test data obtained from the AMCA chamber. The standard k-ε turbulence model is implemented in the simulation and the results show that the model is insufficient in calculating the location of the separation point and the pressure change on the blade surfaces for separated flows. The aerodynamic performance and noise of a bionic fan are optimized by Chen et al.^5^, using Taguchi mass loss function to decrease the noise and increase the mass flow rate. Li^6^ parameterized the influence of blade angle and radial blade angle by using the numerical thermal fluid model verified previously. Wang et al.^7^ combined artificial neural networks and genetic algorithms to optimize the calculation. The calculation results show that the isentropic efficiency and stall margin of the system could be effectively improved by this method. The summary of those studies is shown in Table 1. Literature^8,9^ represent a numerical background in the noise prediction with a CFD procedure, and the second one is a comparison of the turbulence models in the tonal noise prediction, which is a good reference for the noise prediction in the future studies. In addition, the simulation results have been verified by many existing studies, which could provide useful information to complete the optimization^10–13^.

**Table 1:** Summary of related literature.

Literature	Findings
^2^	The performance of axial fans could be effectively improved by blade forward tilt and blade forward sweep
^3^	The turbulent kinetic energy downstream of the fan is highly affected by the sweep
^4^	The standard k-ε turbulence model is insufficient in axial fans simulations
^5^	Taguchi mass loss function was used to decrease the noise and increase the mass flow rate
^6^	The influence of blade angle and radial blade angle was parameterized
^7^	Artificial neural networks and genetic algorithms were combined to optimize the calculation

The parameters of fans are mainly studied in the above research excluding the effect of the guide vane on the air volume flow rate. The front guide vane could make the airflow produce negative pre-rotation contrary to the blade rotation direction, which makes the axial flow of the axial fan produce winding speed, to improve the total pressure of the axial flow fan. When the fluid passes through the blades, it will generate a partial velocity in the circumferential direction, and the rear guide vane can change the flow direction so that the kinetic energy generated by the partial velocity could be converted into pressure energy. It can be concluded that guide vanes are important factors affecting the efficiency of axial flow fans. The best design parameters of guide vanes of an axial flow fan are obtained through the DOE (Design of Experiments) method, which provides a research basis for the optimization of guide vanes of other axial flow fans.

This case study could fill some gaps in guide vanes optimization and the optimal method could provide a reference for the optimization of other types of axial flow fans. In order to get the best performance of the axial fan, the design of guide vanes needs to be modified under different working environments and fan structures. The combination of CFD and DOE methods could reduce the research cost and shorten the research period. After verifying the results of CFD calculation, simulation can replace some experiments and obtain data that is difficult to measure. The DOE method can reduce repeated experiments and achieve the desired effect with the least number of experiments. Therefore, these two methods were adopted to conduct the research to get the optimal design of the guide vane, which is meant for reducing the electricity cost.

## Methods

**Figure 1:**
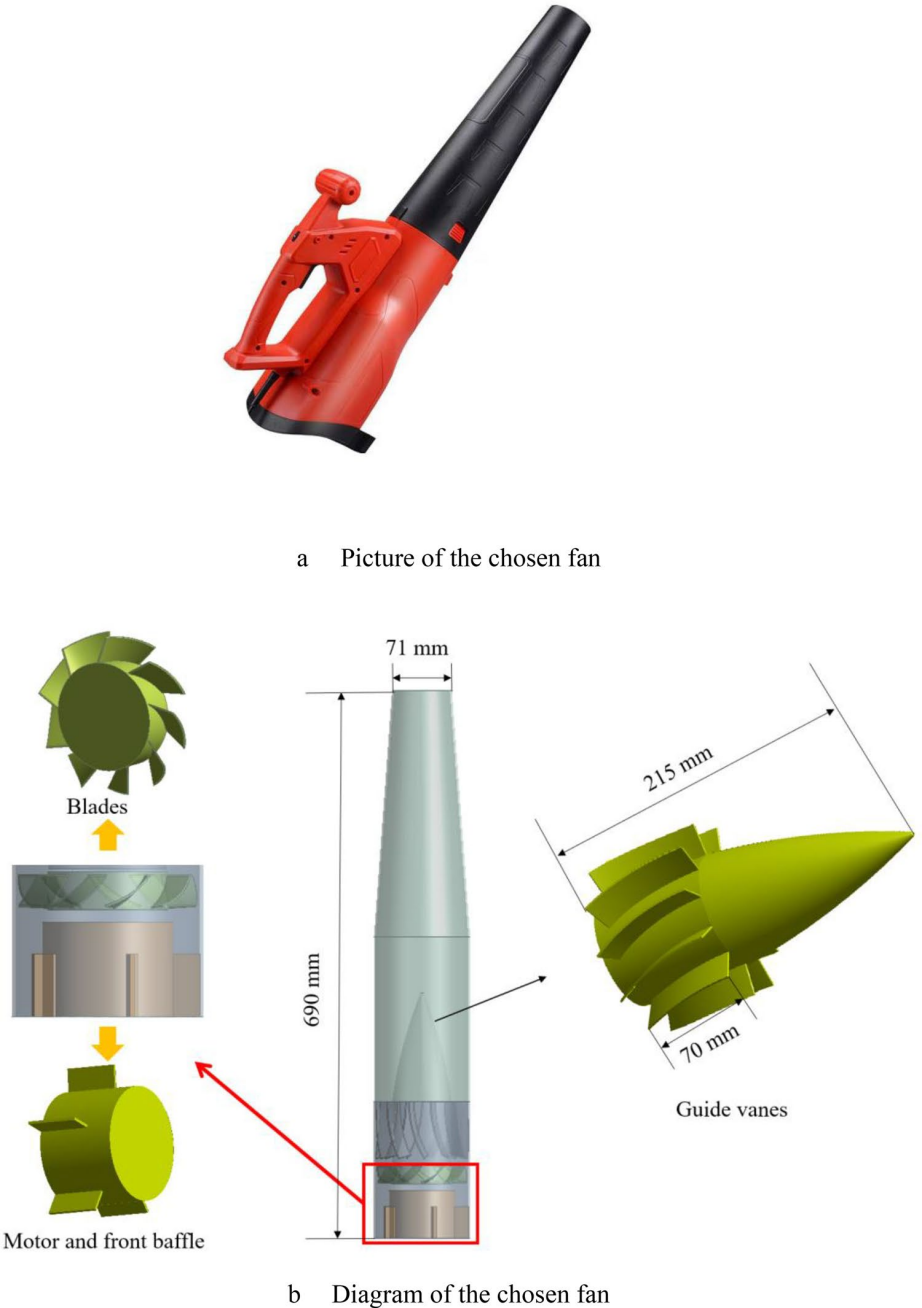
(**a**) Picture of the chosen fan. (**b**) Diagram of the chosen fan.

A small axial flow fan which is used for cleaning the dust was selected. As shown in Fig. 1, the fan has rear guide vanes (11 vanes) and a 9-blade impeller. The duct radius is 117 mm. The blade chord and span of the impeller are 29 mm and 18 mm respectively, and the vane chord and span are 76 mm and 22 mm separately. The rotation speed is set as 5000 r/min, the ambient pressure is 1 atm, and the temperature is 25 °C. The whole fan is modelled and meshed by means of parametric modelling.

The assumptions and simplifications of this paper are as follows,

1. All the wall boundaries in the simulation are no-slip walls.
2. The motor of the fan is simplified and not included in the simulation.

The number and total chord of guide vanes were selected as factors, and the air volume flow rate was selected as the calculation target. The DOE method was used to conduct parameter sensitivity analysis to obtain the influence sequence of input factors, and then the Gaussian Process method was used to obtain the optimal operating point. The above process is described in detail below.

### Modelling and parameter settings of simulations

**Figure 2:**
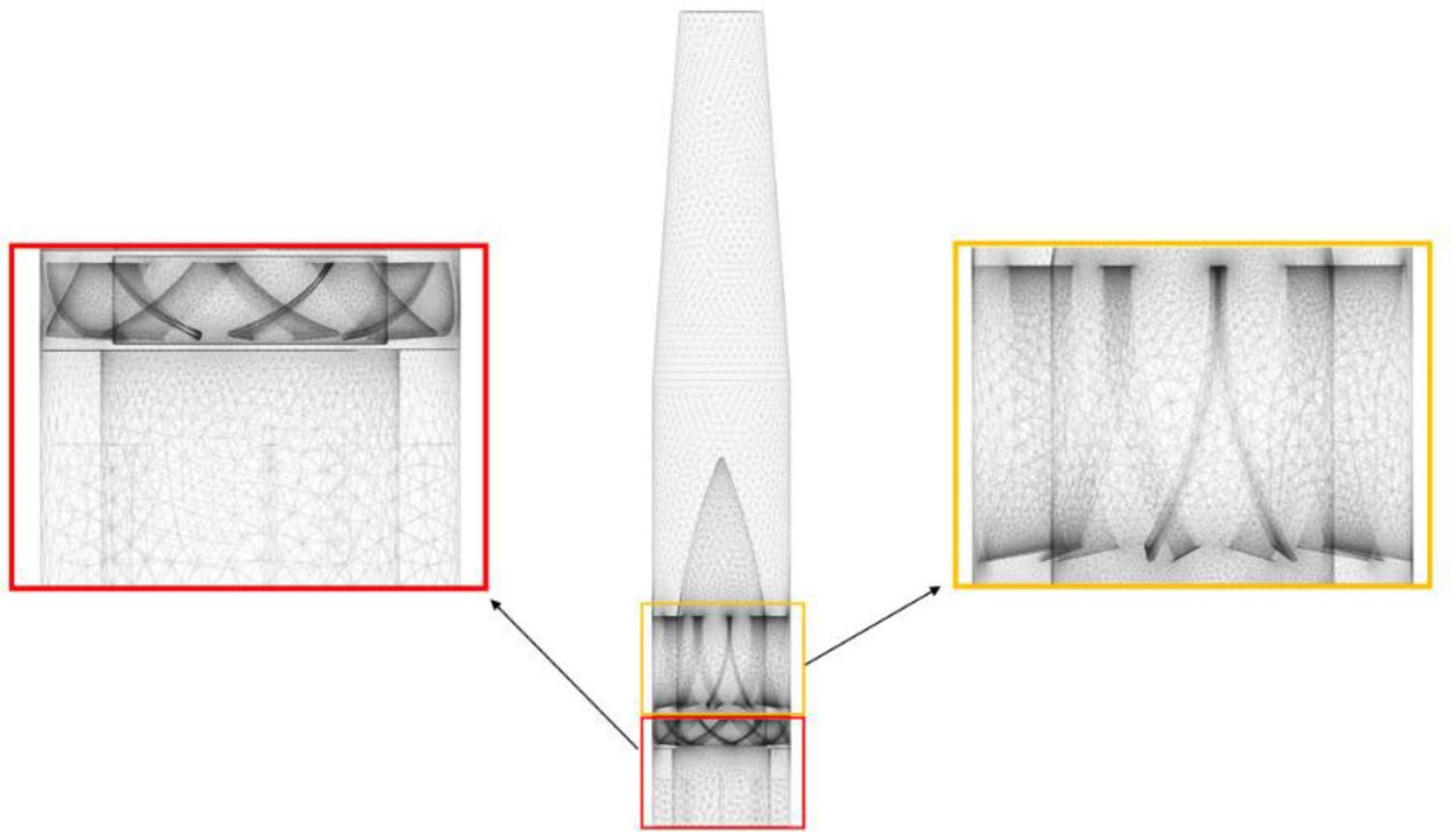
Diagram of mesh.

Parametric modelling is carried out by the Design Modeler in the ANSYS suite, which can efficiently complete the modification of the model. The parameters that need to be changed, such as the number of guide vanes, need to be marked in the geometry-establishing process to facilitate subsequent model changes. ANSYS Meshing is used as the meshing software, which can quickly generate a high-quality unstructured mesh and is very suitable for the mesh partitioning of a large number of geometric models generated by parametric modelling. The mesh is generated as shown in Fig. 2. As shown in this figure, the simulation domain is the internal flow field of the fan. The inlet and outlet boundary are set as pressure inlet and outlet respectively. The steady RANS equations with the SST $k - \omega$ model were chosen as general equations^14^.

**Figure 3:**
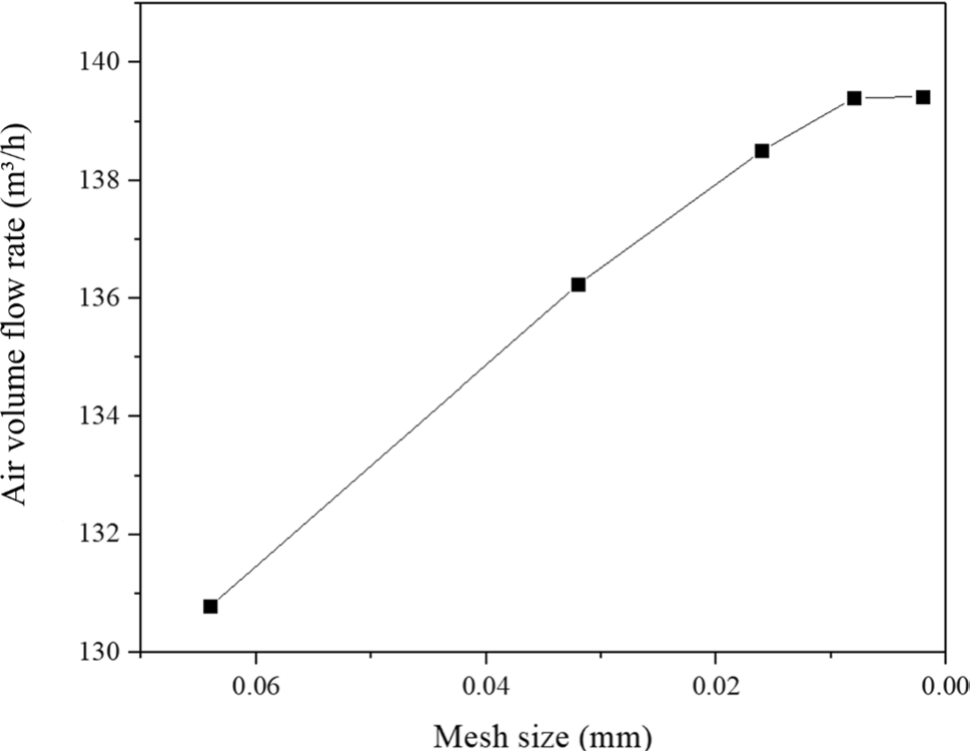
The calculation results of different mesh size.

Next, mesh independence was conducted in order to demonstrate the reference value of the simulation. The mesh is refined by global refinement with the constant mesh size of the first inflation layer, and the Y + value near the wall is about 1 with different global mesh sizes. The mesh data is shown in Table 2. The air volume flow rate of the outlet under different mesh numbers was analysed and summarized, as shown in Fig. 3, and the suitable global mesh size (0.008 m) is obtained and used in the following calculations. The suitable mesh details and quality are shown in Table 3, which shows that the quality of the mesh is suitable for calculation (skewness less than 0.98).

**Table 2:** Mesh information.

Mesh size (m)	Number of cells	Number of nodes
0.064	1.13 × 10^6^	3.0 × 10^5^
0.032	2.73 × 10^6^	6.67 × 10^5^
0.016	5.33 × 10^6^	1.23 × 10^6^
0.008	5.95 × 10^6^	1.35 × 10^6^
0.002	8.06 × 10^6^	2.0 × 10^6^

The size in the table is the default size in ANSYS Meshing rather than the uniform size, the smallest size is 0.0002 m in some particular parts under different mesh sizes.

**Table 3:** Mesh details and quality.

Item	Value
maximum size	1.6 × 10^–2^ m
minimum size	8.0 × 10^–5^ m
minimum skewness	4.7 × 10^–6^
maximum skewness	0.89989
average skewness	0.23723
standard deviation of skewness	0.12432

The solver used is ANSYS Fluent (version 15.0), which is used widely around the world. The numerical scheme used in this solver is the second order upwind scheme. In order to demonstrate the rotor rotation effect, it is necessary to set MRF parameters in the rotation area. The MRF model is the simplest method to calculate variables in the fan rotation domain. By setting the rotation speed in the rotation domain, the transient problem is approximately regarded as a steady-state problem to solve. If the region is at rest, the equation is converted to rest form. At the interface of the computational domain, a local reference frame is used to calculate the flux of flow variables in one area and convert them to adjacent areas. Although the MRF method is an approximate method, it can provide a reasonable computational model in many application scenarios. For example, the contact interface between the rotating region and the stationary region of turbo machinery is relatively simple, and there is no large-scale transient effect between the impellers, so the MRF model can be used.

### The determination of design points based on DOE

In order to improve the reliability of the analysis and reduce the calculation time, the DOE principle is adopted for the design of geometric parameters and subsequent analysis. DOE is a method to study the influence of input parameters on output parameters^15,16^. In OFAT (one-factor-at-a-time), only one-factor changes and the other factors remain the same, so it is intuitive to capture the impact of a single factor in the test area. However, OFAT cannot simulate the interaction between input factors, and the information of the entire design space cannot be captured because of the size of the selected area^17^. DOE has tools for capturing and studying nonlinearity. There are a number of CFD experiments using response surface design and space fill design. Space-filling design is used in this paper due to the deterministic output of the CFD method^18–24^. McKay et al. presented a design method called the Latin hypercube design (LHC)^25^, which is the most common space-filling design. Loeppky et al. found that using 10 times the number of input factors could have a better result, which become a popular guideline now^26^.

The input parameters are the number and chord of guide vanes, and the output parameters are the air volume flow rate of the fan. OFAT experiments could not clarify the mixing effect of two factors on the output. Therefore, we constructed a two-factor LHC and divided the two input parameters into 20 evenly spaced factor levels in the JMP software^27^. The LHC design needs to consider the extreme cases, 3 and 22 are considered after many simulations. The design points are shown in Table 4. According to the previous simulation experience, we set the number of guide vanes in the range of 3–22, and the chord change value of guide vanes in the range of − 70 mm ~  + 120 mm.

**Table 4:** Design points.

Design point number	Number of guide vanes	Total chord change value of guide vane (mm)
1	8	50
2	7	10
3	19	− 20
4	22	110
5	9	− 10
6	13	− 70
7	16	100
8	11	80
9	14	− 30
10	6	90
11	12	20
12	18	30
13	21	− 60
14	20	70
15	10	− 40
16	4	40
17	15	60
18	17	0
19	5	− 50
20	3	120

### The Gaussian process method used in results analysis

In order to find the optimal design parameters, a proxy model for Latin hypercube space-filling design is established by the Gaussian Process model. Unlike low-order polynomial models, which define the form of the model before analysis, Gaussian Process models are flexible and could be adapted to complex surfaces. In addition, Gaussian Process models are interpolative, and the results are fully consistent with experimental observations^24^. Sacks et al.^28^ developed the Gaussian Process model in 1989 and the formulas are shown as follows:

1 $$ y = \mu + z{(}{\mathbf{x}}{),} $$

2 $$ r_{ij} = e^{{ - \sum\limits_{k = 1}^{n} {\theta_{s} (x_{ik} - x_{jk} )}^{2} }} , $$

in which $z{(}{\mathbf{x}}{)}$ is a normal random process with covariance $\sigma^{2} {\mathbf{R}}$.$r_{ij}$ is the correlation coefficient. $\theta_{s}$ (weight), $\sigma$ (population standard deviation) and $\mu$ (average) are the model parameters to be estimated, the prediction equation is

3 $$ \hat{y}({\mathbf{x}}) = \hat{\mu } + {\mathbf{r^{\prime}}}({\mathbf{x}}){\mathbf{R}}(\hat{\theta })^{ - 1} ({\mathbf{y}} - {\mathbf{j}}\hat{\mu }), $$

where $\hat{\theta }_{s}$, $\hat{\sigma }$ and $\hat{\mu }$ is the maximum likelihood estimation of $\theta_{s}$, $\sigma$ and $\mu$ respectively. In addition,

4 $$ {\mathbf{r^{\prime}}}({\mathbf{x}}) = {[}{\mathbf{r}}{(}{\mathbf{x}}_{{\mathbf{1}}} ,{\mathbf{x}}{)},{\mathbf{ r}}{(}{\mathbf{x}}_{{\mathbf{2}}} ,{\mathbf{x}}{)}, \, \ldots \, ,{\mathbf{r}}{(}{\mathbf{x}}_{{\mathbf{n}}} ,{\mathbf{x}}{)],} $$

which contains the design point vectors. The validation of Eqs. (1)–(4) can be found in the literature^29^.

## Experimental validation

**Figure 4:**
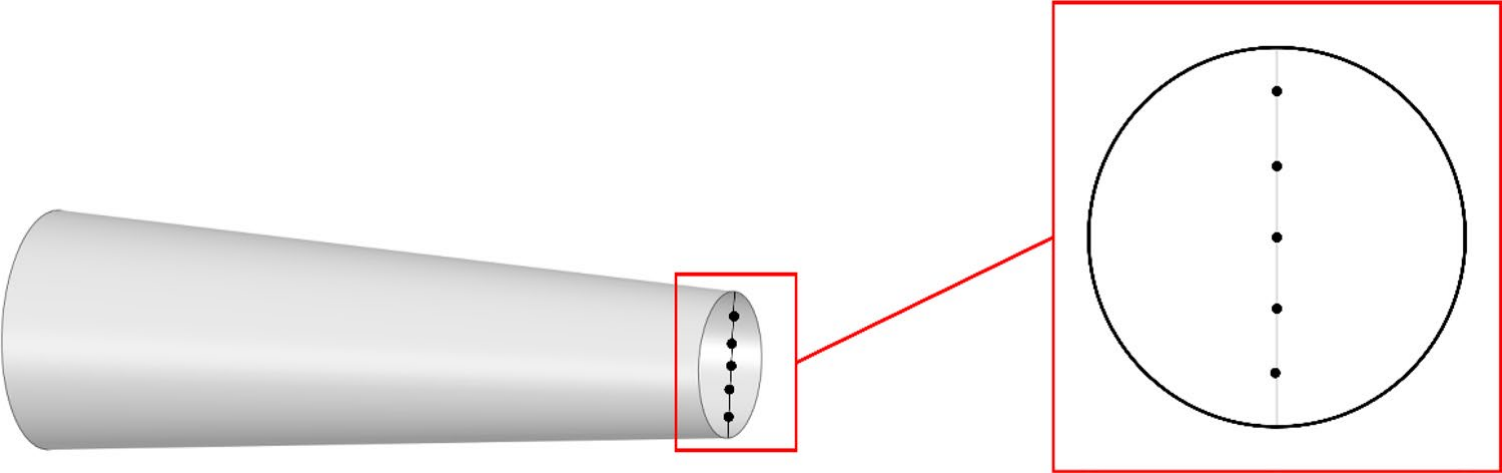
Measuring points.

**Figure 5:**
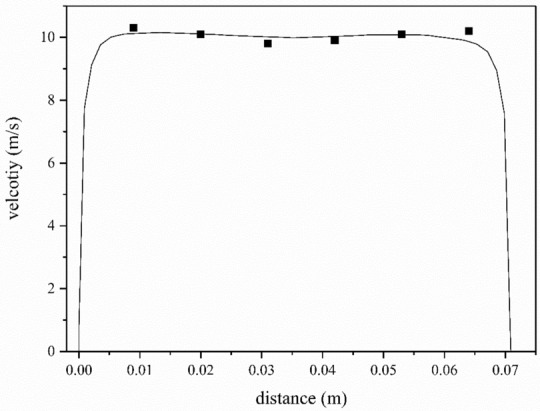
The comparison of the experimental and simulations results. The velocity of measuring points was obtained by pitot tube, which has a similar trend with the calculation results.

In order to ensure the validity of the simulation results, the original fan was simulated and verified by experiments. The air velocity is collected at the centre of the outlet, which is measured through the pitot tube. The experimental velocity is 7.5–11.3 m/s (average 9.4 m/s), and the simulated velocity is 9.8 m/s at 5000 r/min. Besides, more points were selected as shown in Fig. 4, the compared results were shown in Fig. 5 and the error tables are shown in Table 5, which shows that the simulation results could be referred to and analysed.

**Table 5:** Error tables.

Distance (m)	Velocity (m/s)	
	Experimental results	Simulation results
0.01	10.3	10.1369
0.02	10.1	10.0965
0.03	9.8	10.0122
0.04	9.9	10.0059
0.05	10.1	10.0812
0.06	10.2	9.98542

## Results and analysis

### Analysis of LHC experimental design results

**Figure 6:**
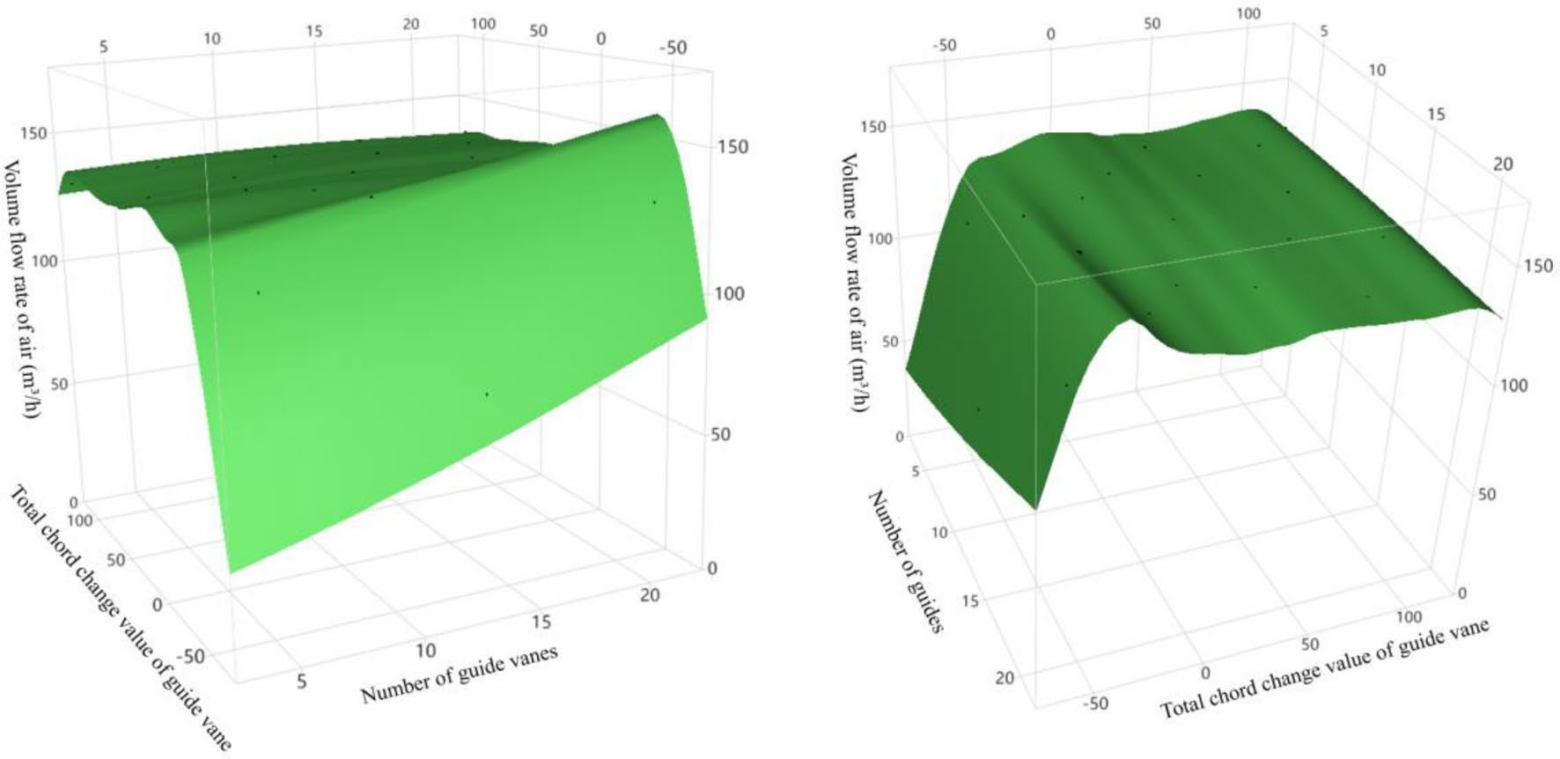
Surface diagram by Gaussian Process method.

The calculation results plotted by the Gaussian Process method are shown in Fig. 6. First of all, most areas of the surface are relatively flat, and only the edge region fluctuates greatly, especially the region where the chord change of the guide vanes changes to a negative value. In addition, the highest point of the whole surface appears in the edge region, which means that the best values may be found outside the surface. Therefore, another prediction needs to be conducted.

**Figure 7:**
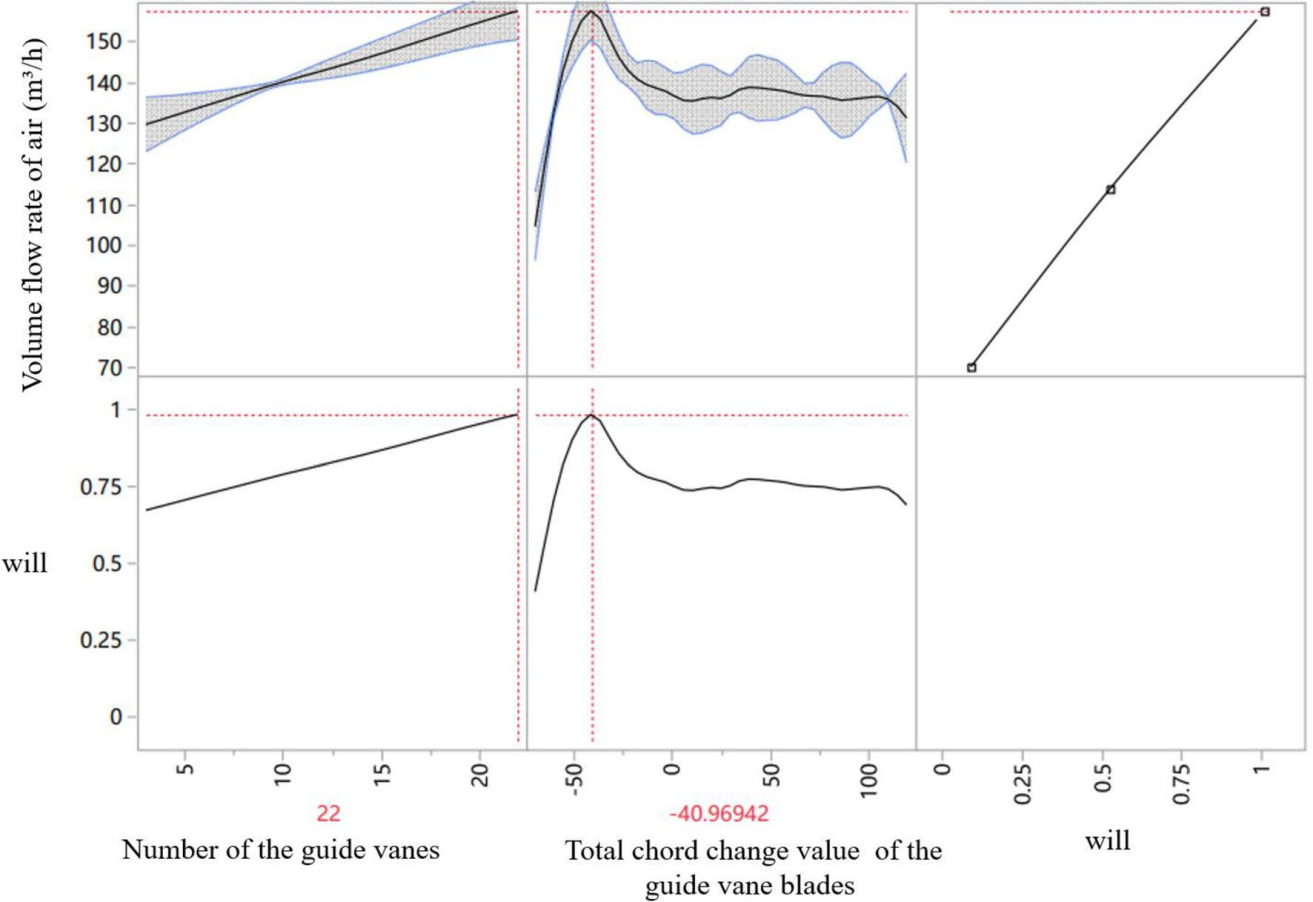
Extreme point prediction curve (first prediction). The air volume flow rate is highest when the chord changed value is − 40.97 mm and the blade number is 22. The grey zone contains the possible results. The no-represented parameter in the first curve (left) is presented in the second one, which means the change of blade numbers on the air volume flow rate is under the same chord change value, − 40.97 mm. And the no-represented parameter in the second curve is presented in the first one. *Note*: Will means a degree of satisfaction (The closer the value is to 1, the better the result).

In order to verify the rationality of the prediction, the extreme point is obtained through the prediction graph (Fig. 7). It could be found in the figure the longer the guide vane chord, the higher the loss along the pipe. However, if guide vanes are too short, they will be unable to correct the deflected airflow, resulting in low air volume.

**Figure 8:**
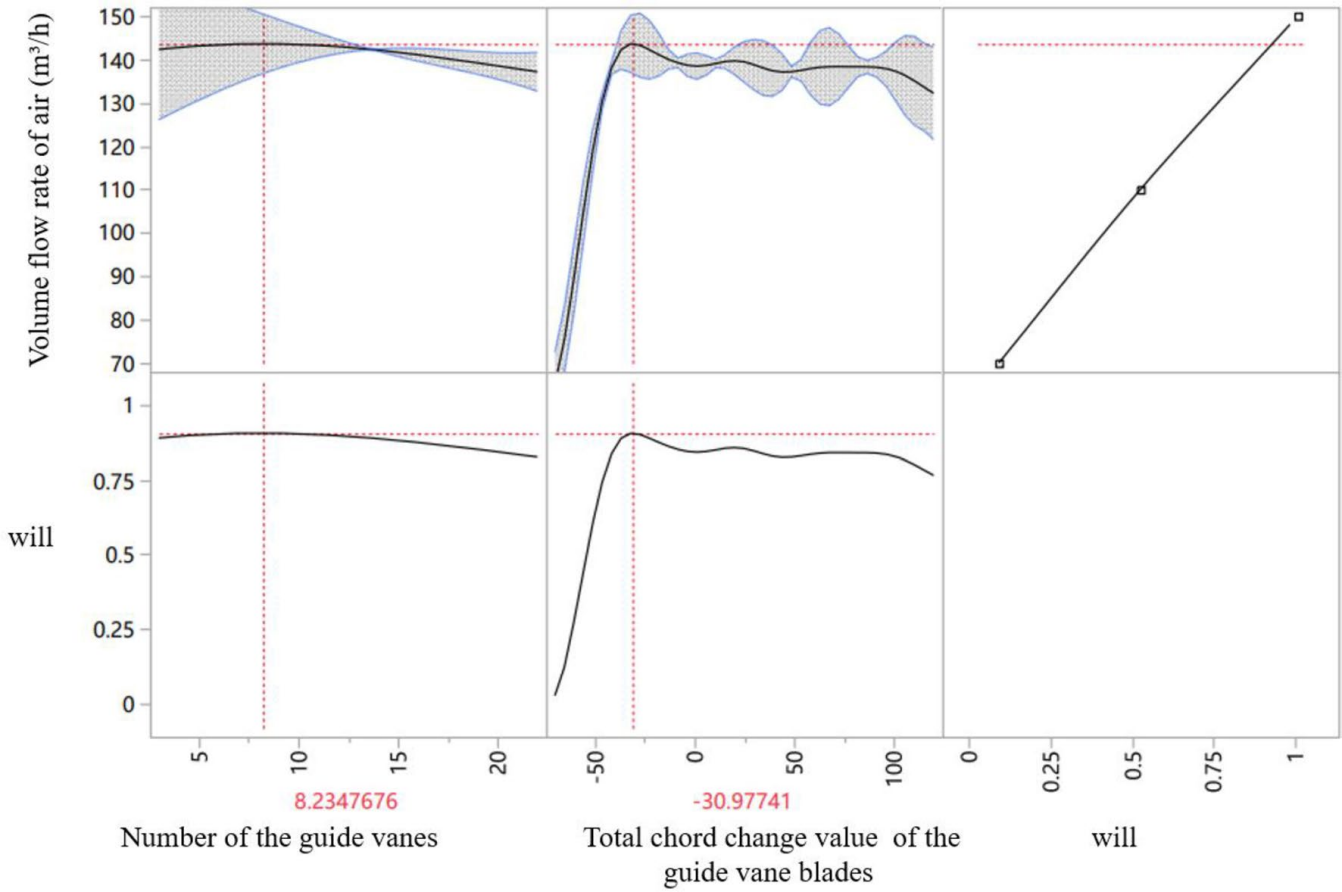
Extreme point prediction curve (second prediction). The air volume flow rate is highest when the chord changed value is − 30.98 mm and the vane number is 8.

**Figure 9:**
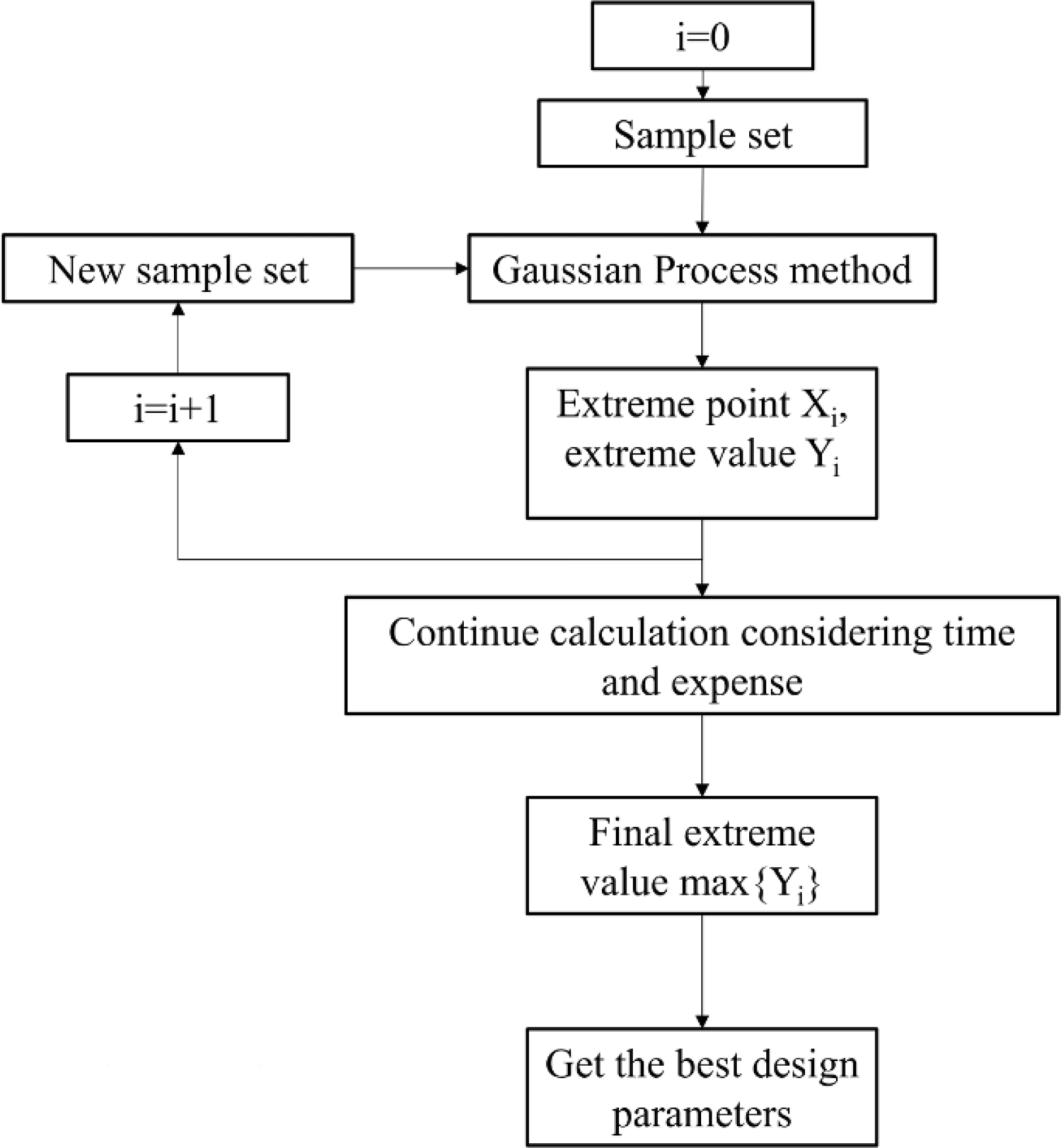
Extreme point search method.

The extreme point in Fig. 7 is taken as a new sample point to be put into the original data set for prediction. Using the Gaussian Process method, the new extreme point is obtained, as shown in Fig. 8. In this figure, the maximum value appears in the inner region of the surface, which can prove that the best value is within the selected range. However, the occurrence of a new extreme point indicates that the previous prediction is not accurate enough, so the following extreme point search method is adopted, as shown in Fig. 9.

### Optimal parameter determination

After several times of predictions by inserting new sample points, it could be found that the air volume flow rate is quite considerable when the guide vanes chord is reduced by 20–40 mm, and the number of guide vanes is determined to be 15–22. After comprehensive consideration of calculation time and expense, the optimal design point in this paper is determined as DP [− 39.0, 18], which means that the chord change value and the number of guide vanes are − 39 mm and 18 respectively, and the air volume flow rate is 142.07 m^3^/h when the rotation speed is 5000 r/min.

### Influence of chord change value and number of guide vanes on air volume flow rate

**Figure 10:**
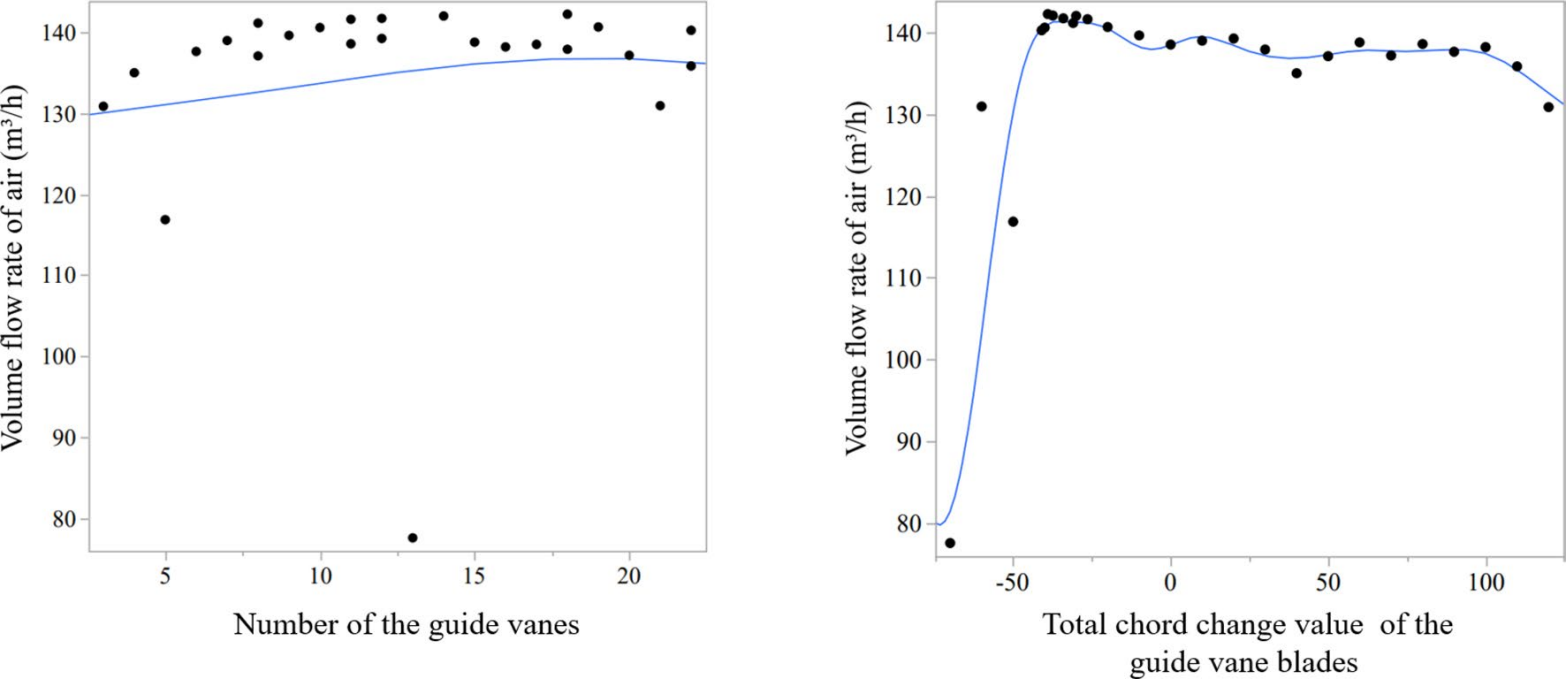
The edge model graph of design points. The black points are simulation results of different design points, and the blue lines are predicted results. The un-represented parameter in each figure is not a constant. The edge model is obtained by putting the design points and the simulation results together in each figure directly.

The edge model graph and model report of design points are shown in Fig. 10 and Table 6 respectively. It could be seen that the guide vane chord plays a leading role in the influence of air volume, and it does have interactions with another parameter.

**Table 6:** Model report.

Inputs	Total sensitivity	Main effect	Interaction
Number of the guide vanes	0.1722364	0.0297873	0.142449
Total chord change value of the guide vane	0.9702127	0.8277636	

**Figure 11:**
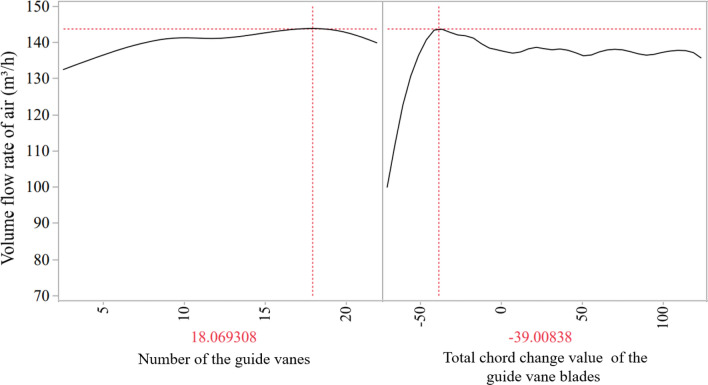
The curves of the inputs on output at the optimal point.

**Figure 12:**
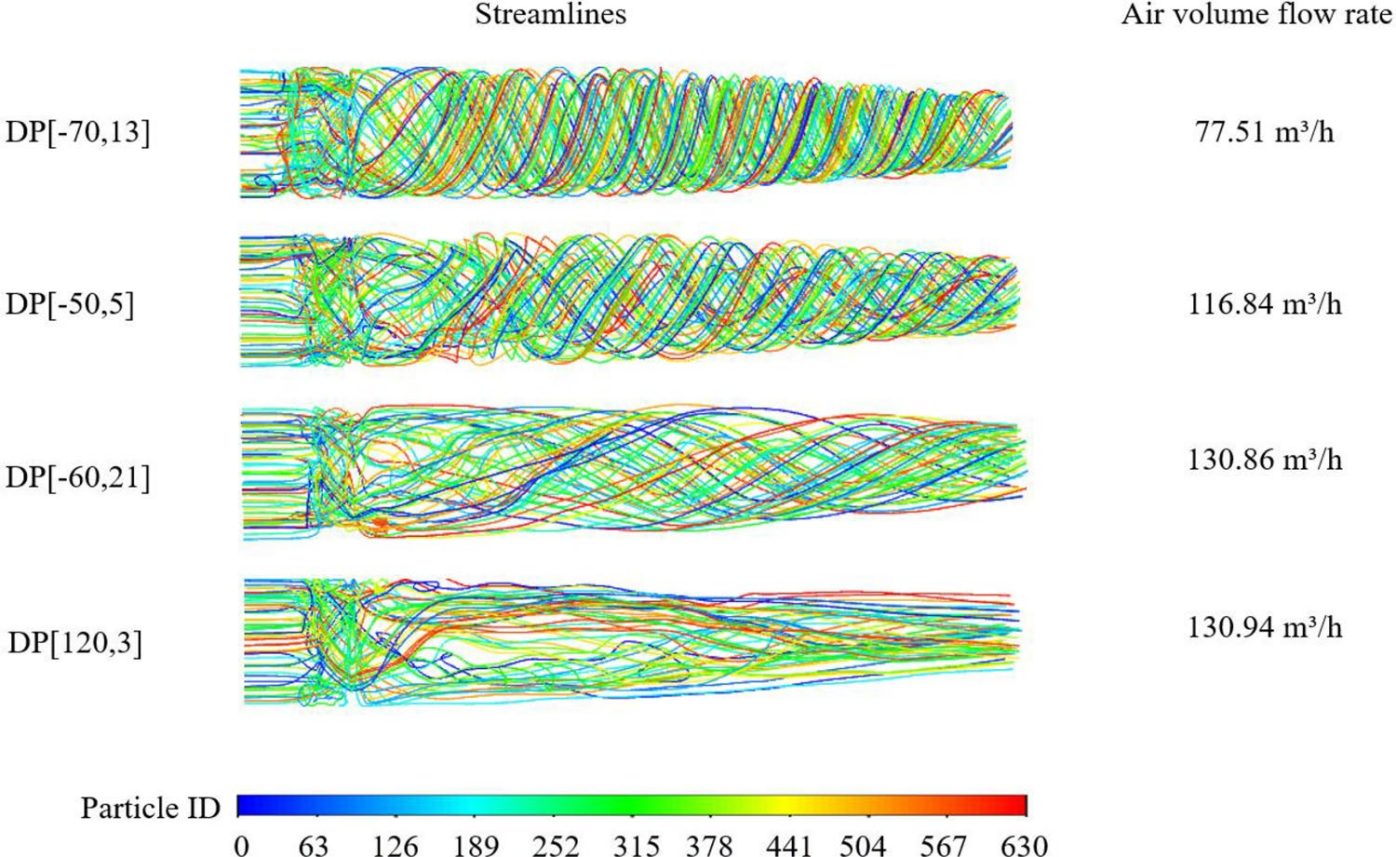
The pathlines of four selected design points.

It could be seen in Fig. 11 that under the optimal number of vanes, the increase of the chord of guide vanes first increases and then decreases the air volume flow rate. Figure 12 is obtained by post-processing the four design points with smaller air volume flow rates, which shows that when the chord of the guide vanes is not enough to correct the deflection of the airflow, the airflow collision in the air duct will increase significantly (DP [− 70,13] and DP [− 60,21]). It could be also found in Fig. 11 that the shape of path lines is related to the air volume flow rate, which shows the importance of the guide vanes design for the axial fans. However, longer guide vanes may cause more resistance loss along the path, resulting in reduced air volume flow rate.

**Figure 13:**
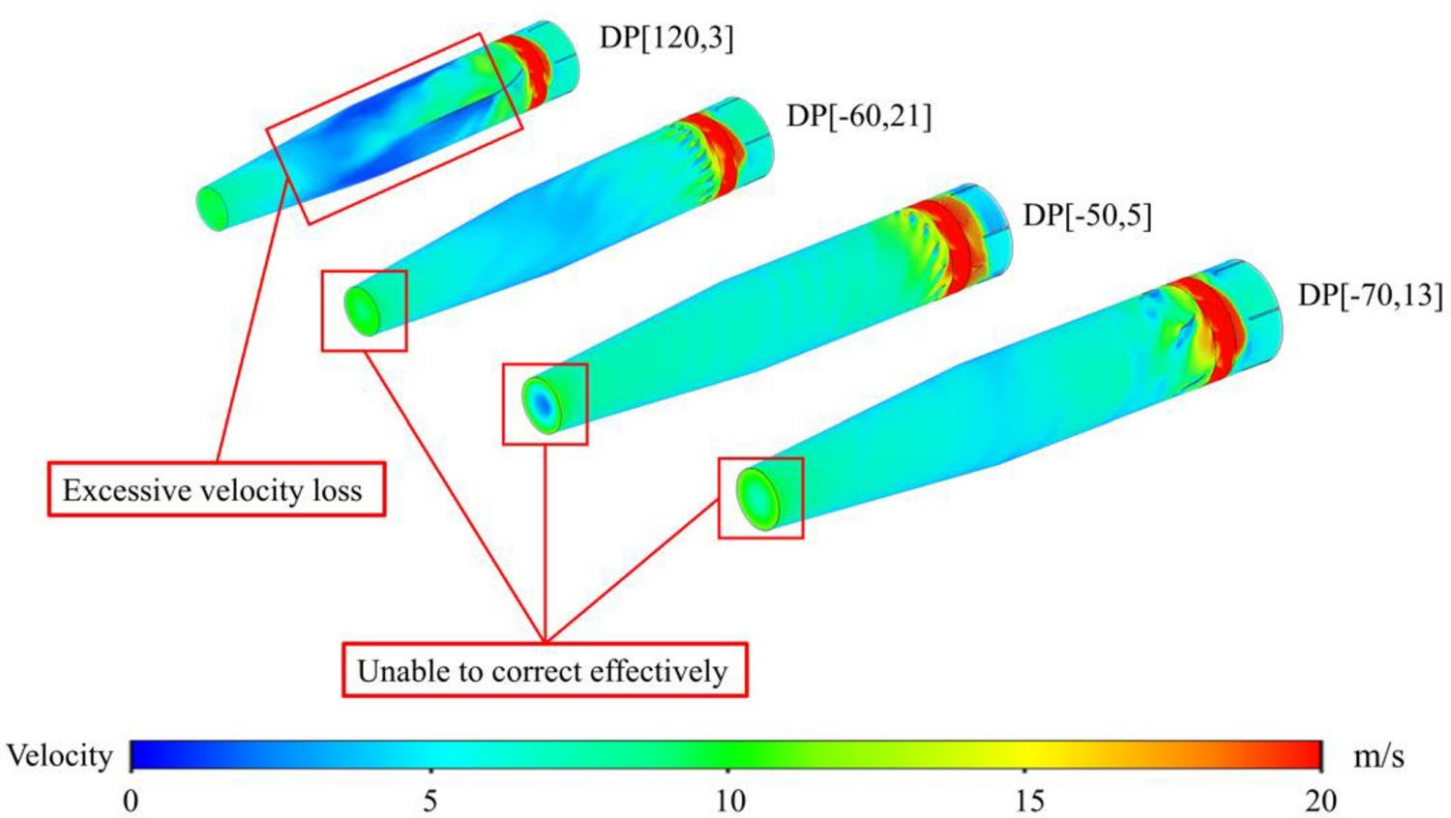
The velocity distribution of selected design points.

The number of guide vanes also has a certain influence on the air volume flow rate. The guide vanes cannot correct the deflection of the airflow well when the number of the vanes is too small (DP[120,3]). But the space for airflow through the guide vanes will be reduced when the number of the vanes is too big, resulting in the high speed of the airflow and more resistance losses along the path, as shown in Fig. 13.

## Conclusions

Various design points are calculated and analysed to find the optimal design parameters by the Gaussian Process method. Under 5000 r/min, the effects of various parameters on the air volume flow rate are analysed. Our conclusions can be summarized as follows.

1. The DOE principle could provide a convenient method to identify the relationship between inputs and outputs in the optimal process of guide vanes;
2. The chord of guide vanes is the main factor affecting the air volume flow rate, while the number of the vanes is not;
3. The optimal study shows that lessening the chord of vanes by 38 mm and increasing the number of vanes to 18 could get better results.

The results presented in this paper could provide a reference optimization method for guide vanes. However, our experiments and simulations are only conducted on the selected axial fan.

In the future, more types of axial fans will be studied and the design of the guide vane will be summarized to form more comprehensive optimization recommendations. In addition, the optimization method will be improved by referring to more advanced methods. All the studies will provide more useful information for the optimization of the guide vane.

## Data Availability

The datasets generated and/or analysed during the current study are available from the corresponding author on reasonable request.

## List of symbols

$\vec{U}$: The average velocity
$G_{k}$ and $G_{\omega }$: Turbulence kinetic energy $k$ and specific dissipation rate $\omega$
$k$: Turbulence kinetic energy
$Y_{k}$ and $Y_{\omega }$: The dissipation of $k$ and $\omega$ due to turbulence
$D_{\omega }$: The cross-diffusion term
$S_{k}$ and $S_{\omega }$: The source terms
$G_{b}$ and $G_{\omega b}$: The buoyancy terms
$G_{k}$ and $G_{\omega }$: Turbulence kinetic energy $k$ and specific dissipation rate $\omega$
$$z{(}{\mathbf{x}}{)}$$: Random process
$$r_{ij}$$: Correlation coefficient
y: The unknown performance function
${\mathbf{R}}^{m} \to {\mathbf{R}}$: Objective function or constraint function
**x**: The design variables
**S**: Design solutions
**y**_*s*_: Function response values
$\hat{y}({\mathbf{x}})$: The agent model
**y**: The known sample function response values
${\mathbf{f}}(x)$: The basis function of known points
$z(x)$: A static stochastic process with zero mean and variance of $\sigma^{2}$
*x*,* w*: Points in space
$R(x,w)$: The correlation function
**F**: The matrix of basis functions of known points
**R**_*i,j*_: The covariance matrix of the points
**r**(*x*): The covariance matrix of the unknown points and the known points
**Z**: The error of the known points
*z*: The error of the unknown points

## Greek

$$\rho$$: Density (kg/m^3^)
$\omega$: The specific dissipation rate (the ratio of turbulence dissipation $\varepsilon$ to turbulent kinetic energy $k$)
$\Gamma_{k}$ and $\Gamma_{\omega }$: The effective diffusivity of $k$ and $\omega$
$$\sigma^{2} {\mathbf{R}}$$: Covariance
$$\theta_{s}$$: Weight
$\sigma$: Population standard deviation
$$\mu$$: Average
$\hat{\theta }_{s}$, $\hat{\sigma }$ and $\hat{\mu }$: The maximum likelihood estimation of $\theta_{s}$, $\sigma$ and $\mu$ respectively
${{\varvec{\upomega}}}$: The weighting factor
${{\varvec{\upbeta}}}$: The weight of the basis function
$$\sigma^{2}$$: The variance
$${{\varvec{\uplambda}}}$$: The eigenvalue
$${\hat{\mathbf{\lambda }}}$$: $${\hat{\mathbf{\lambda }}} = - \frac{{{\varvec{\uplambda}}}}{{2\sigma^{2} }}$$
$${{\varvec{\upbeta}}}^{*}$$: $${{\varvec{\upbeta}}}^{*} = ({\mathbf{F}}^{T} {\mathbf{R}}^{ - 1} {\mathbf{F}})^{ - 1} {\mathbf{F}}^{T} {\mathbf{R}}^{ - 1} {\mathbf{y}}$$
$${{\varvec{\upgamma}}}^{*}$$: $${{\varvec{\upgamma}}}^{*} = {\mathbf{R}}^{ - 1} ({\mathbf{y}} - {\mathbf{F\beta }}^{*} )$$

## Subscripts

DOE: Design of experiments
CFD: Computational fluid dynamics
LHC: Latin hypercube design
RNG: Renormalization-group
MRF: Multiple reference frame
DP: Design point
